# Computational Intelligence Techniques for Tactile Sensing Systems

**DOI:** 10.3390/s140610952

**Published:** 2014-06-19

**Authors:** Paolo Gastaldo, Luigi Pinna, Lucia Seminara, Maurizio Valle, Rodolfo Zunino

**Affiliations:** Department of Electric, Electronic, Telecommunication Engineering and Naval Architecture, DITEN, University of Genoa, Via Opera Pia 11a, 16145 Genova, Italy; E-Mails: luigi.pinna@unige.it (L.P.); lucia.seminara@unige.it (L.S.); maurizio.valle@unige.it (M.V.); rodolfo.zunino@unige.it(R.Z.)

**Keywords:** electronic skin, touch modalities, pattern recognition, computational intelligence, human-robot interaction

## Abstract

Tactile sensing helps robots interact with humans and objects effectively in real environments. Piezoelectric polymer sensors provide the functional building blocks of the robotic electronic skin, mainly thanks to their flexibility and suitability for detecting dynamic contact events and for recognizing the touch modality. The paper focuses on the ability of tactile sensing systems to support the challenging recognition of certain qualities/modalities of touch. The research applies novel computational intelligence techniques and a tensor-based approach for the classification of touch modalities; its main results consist in providing a procedure to enhance system generalization ability and architecture for multi-class recognition applications. An experimental campaign involving 70 participants using three different modalities in touching the upper surface of the sensor array was conducted, and confirmed the validity of the approach.

## 1. Introduction

Electronic skin enables robots to sense their surroundings through touch. In this sense, robots represent an ideal stimulus to establish a controlled interaction with humans in a real world environment, making it possible to study both the cognitive and physical aspects of the robot-environment interaction. To enable the robot to grasp and manipulate objects, touch sensors can be integrated into the hands (e.g., [1–3]). Hands and upper arms are covered with the artificial skin to touch-triggered withdrawal reflexes [4], while tactile sensors specifically integrated on the arms can be used, for example, to indicate position adjustments [5]. To improve robot-environment interaction, other parts of the robot body can be covered with tactile sensors, e.g., the hands, the arms, the cheeks, the feet and the torso.

Nevertheless, reliable tactile systems are still an open issue as many technological and system issues remain unresolved and require a strong interdisciplinary effort to be addressed effectively. Technologies for effective signal transduction involve both materials and electronics aspects. However, as the overall performance depends on how the different building blocks are integrated, research on system issues has to be coupled to transducer development. In particular, as in the human perceptual mechanism a number of components of the sensory system manage information coming from the large number of skin receptors [6], the effective utilization of tactile sensors requires research attention towards issues like deciphering the information contained in tactile data [7]. Therefore, the design of a tactile sensing system should also include effective methods for the interpretation of sensor data. Such aspect is crucial in that sensor data typically support the recognition of either certain properties of the contact surfaces or certain qualities/modalities of touch.

Pattern-recognition methods proved to be effective in specific tasks such as materials classification and/or recognition of materials textures, patterns, shapes, hardness and size (e.g., [8–12]). However, few works report on the classification of touch modalities and gestures [13–15]. Suitable computational models are required to accomplish these goals. Contact materials have been recognized by Support Vector Machine (SVM), Regularized Least Square (RLS) and Regularized Extreme Learning Machine (RELM) [8], feature extraction from sensory data using self-organizing maps (SOMs) is reported in [16], neural network algorithms applied on tactile data have been used to obtain specific surface features of the contact object [17] and Bayes trees allow one to distinguish different materials from their surface texture [18].

**Figure 1.**
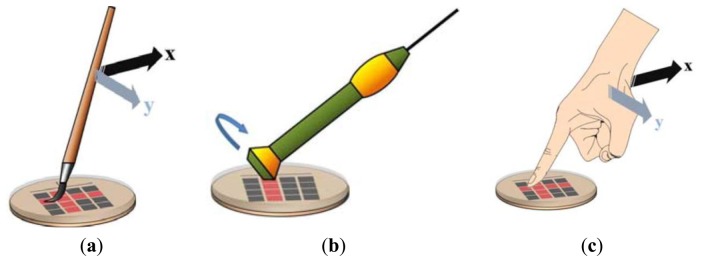
Touch modalities. (**a**) Paintbrush brushing; (**b**) finger sliding; (**c**) washer rolling.

Touch-modality recognition is the specific sensorial problem that is tackled in this paper. The skin surface is subject to a variety of possible stimuli, and the system is expected to discriminate the various modalities of physical interaction. The problem complexity stems from the bi-dimensional sensing structure, which is augmented by the time-varying distribution nature of the stimulus and pressure pattern. Figure 1 illustrates the problem setting by showing three touch modalities, namely, sliding the finger, brushing a paintbrush and rolling a washer.

The application of ML techniques to touch-modality recognition showed that the processing of sensor data under a tensor-based representation could yield promising recognition performance without a significant increase in complexity [19]; that work mostly proved the specific advantages of tensor-based sensor processing over a conventional, vector-based representation of raw data.

The research presented in this paper tackles the critical issue of performance evaluation and reliability assessment of the tensor-based approach for the accurate classification of touch modalities, with specific attention paid to the generalization ability of the deployed methods, and the possibility to address realistic recognition problems. The theoretical framework introduced in [20] is used to derive a ML-based system for pattern recognition that deals with the interpretation of touch modalities and is specifically designed to treat tensor signals. The novelty of the proposed approach, therefore, consists both in characterizing the tensor-based paradigm in terms of expected performance from a quantitative viewpoint, and in applying ML techniques in multi-class recognition domains

In this work, tactile data have been acquired by an electronic skin based on a piezoelectric sensor array. An experimental campaign involving 70 participants has been conducted to employ the tensor-based pattern-recognition system for the classification of touch modalities in three different bi-class classification problems and in the 3-class classification problem involving all three touch modalities.

This paper is organized as follows: Section 2 describes the tactile sensing system, which includes the sensor array based on polyvinylidene fluoride (PVDF) sensing elements, the interface electronics and the data acquisition and processing. Section 3 illustrates the theory of the kernel-based algorithm to deal with tensor data, while Section 4 suggests a practical model-selection procedure. Section 5 describes the experimental campaign and discusses experimental results. Concluding remarks are contained in Section 6.

## 2. Tactile Sensing System Based on Piezoelectric Transducers

Though the only requirement for the sensor array is to provide *tensor* signals to be managed by the proposed ML-based system, in this paper the method is specifically demonstrated with an electronic skin based on *piezoelectric* transducers. In the following, the properties of the sensing material introduce the description of the tactile acquisition system.

### 2.1. The Sensing Material

A large number of daily tasks involve dynamic contacts and hence it is desirable to have an electronic skin which is responsive to a wide range of mechanical stimuli (in humans this range is approximately 0–1 kHz). While static and quasi-static contact events are usually managed by capacitive tactile elements, piezoelectric technology is suitable for detecting dynamic contact events. Piezoelectric polymers are particularly interesting in that they are mechanically flexible, conformable, and exhibit wider frequency bandwidth [21,22]. Further, they are low-cost, can be prepared in thin films and can be cut into any desired shape [23].

The electromechanical response of piezoelectric polymers can be recorded either in the form of charge generation or in form of a change in capacitance [24]. Accordingly, they can be easily manufactured and integrated with flexible PCB and electronic interface circuitry can be easily developed using off-the-shelf electronics. In particular, polyvinylidene fluoride (PVDF) has been chosen as piezoelectric polymer to build the sensor array. The PVDF film was a circular portion (diameter = 7 cm) of a commercial foil from MEAS—Measurement Specialties Inc. (Hampton, VA, USA).

### 2.2. The Tactile Acquisition System

To fabricate an electronic skin system based on piezoelectric transducer arrays, issues concerning the manufacturing technology, the interface electronics and the system integration have to be addressed. PVDF must be first integrated into structures which also include a substrate and a protective layer. The piezoelectric film is glued to a flexible printed circuit board (PCB) structure which is conformable and flexible, covered by an elastic layer to protect the sensor from physical damage or chemical contamination.

**Figure 2.**
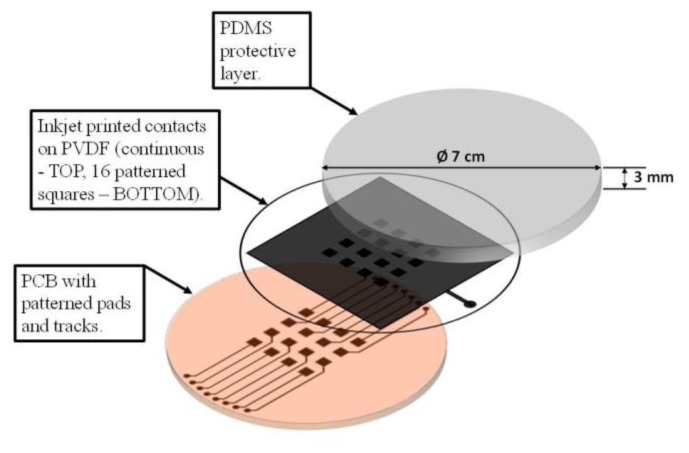
Tactile sensor made of an array of piezoelectric polymer transducers.

**Figure 3.**
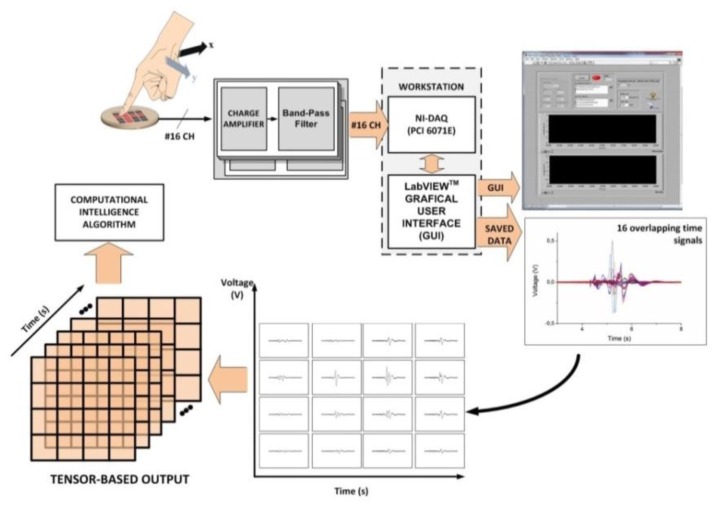
Scheme of the tactile acquisition system. Focus is on the tensor representation of output data.

In order to build the sensor array (Figure 2), the piezoelectric film features *ad hoc* metal contacts (16 square electrodes on the PVDF lower surface and a ground layer on the PVDF top), which are deposited by inkjet printing [25]. The underlying PCB substrate is provided with metal electrodes and tracks to extract the lower PVDF signals. Once the PVDF film has been glued on the PCB substrate, a polydimethylsiloxane (PDMS) 2 mm thick elastomer layer is directly integrated on top [24]. Figure 3 shows a scheme of the overall tactile acquisition system.

The mechanical-to-electrical transduction by each PVDF taxel is measured as a generated charge and converted to a voltage by a 16-channel charge amplifier. It includes a charge amplifier (CA) [26] cascaded with a band pass filter (BPF) featuring a bandwidth from 0.3 Hz to 1.5 kHz. The bandwidth of the CA + BPF is 2.5 Hz–1.5 kHz.

The subsequent step is to acquire the tactile system output data both to be visualized by the operator and to be processed by the pattern-recognition system for touch modality classification. For this aim, output signals are acquired at 3 k Samples per second by a DAQ board (NI PCI-6071E) and visualized by a LabVIEW™ graphic user interface (GUI) in the time domain.

As the *time*-varying tactile interaction is conveyed through the skin protective layer to the 2D geometry of the sensor array, it seems that a touch-modality aware sensing framework should involve a tactile hardware that can yield a *tensor* signal. This morphology of the tactile signal includes both time-varying features that actually contribute to determine the touch modality (e.g., contact pressure on the electronic skin, stimulus duration, *etc.*) and the sensor spatial arrangement. The following sections illustrate an approach to the management of tensor-like data for extracting directly meaningful information about the original mechanical stimulus.

## 3. Machine Learning for Touch Modality Recognition

Several works in the literature adopt Machine Learning (ML) algorithms for pattern-recognition tasks in tactile sensing systems (e.g., [8,27–30]). The rationale is that ML techniques can support predictive systems that make reliable decisions on unseen input samples [31]. This ability is especially appealing in the case of the interpretation of sensor data, as complex, non-linear mechanisms characterize the underlying phenomenon to be modeled and an explicit formalization of the input-output relationship is difficult to attain. ML technologies model the input-output function by a “learning from examples” approach; eventual implementations can vary according to different application scenarios, but all share a common probabilistic setting.

Tactile data are first processed by feature-extraction and transformed into a multi-dimensional vector to feed the learning algorithm. The ability of the feature space to characterize the underlying perceptual phenomenon is crucial to the effectiveness of the whole pattern-recognition task. The feature-extraction process, though, may bring about the loss of some structural information embedded in the original structure of the tactile data. The literature already proved [20,32,33] that tensors provide an efficient tool to describe multidimensional structured data, and that the corresponding learning methods can favorably exploit the *a priori* information of data structure to achieve satisfactory generalization abilities. The theoretical framework introduced in [20] allows one to extend every learning machine based on kernel methods to a tensor-based learning model. Such feature is attractive in that kernel methods support both supervised paradigms (*i.e.*, learning schemes that address classification problems) and unsupervised paradigms (*i.e.*, learning schemes that address clustering problems). This in turn may provide an effective tool in the specific case of touch recognition, as the inherent complexity in discriminating classes of gestures/modalities that are often overlapping may hinder a straightforward implementation of supervised learning tools. This aspect will actually be discussed in more details in Section 4. The following Section 3.1 will deal briefly with kernel methods for ML-based pattern recognition. Section 3.2 will discuss the theoretical framework that allows one to extend kernel methods to tensor data.

### 3.1. Kernel Methods for Pattern Recognition and Tensor-Based Representation

The empirical learning of a generic mapping function *γ* stems from a training procedure that uses a dataset, **X**, holding *N_p_* patterns (samples). In a binary classification problem, each pattern includes a data vector, **x** ∈ ℜ*^n^*, and its category label *y* ∈ {−1, 1}. When developing data-driven classifiers, the learning phase requires both **x** and *y* to build up a decision rule. After training, the system processes data that do not belong to the training set and ascribes each test sample to a predicted category, *ŷ*. The function that predicts the class of a sample is a sharp decision function, *ŷ* = *sign*(*f*(**x**)), where *f*(**x**) is expected to effectively approximate the ‘true’ mapping function *γ*.

In pattern-recognition technologies, the class of kernel methods embeds the techniques that express *f*(**x**) as a weighted sum of some nonlinear “kernel” basis functions. Generalization ability relies on two main concepts: the function *f*(**x**) belongs to a reproducing kernel Hilbert space (RKHS), and regularization theory is used as the conceptual basis [31]. The former concept—in practice—means that kernel classifiers benefit from the so-called kernel trick [31]: patterns **x***_i_* and **x***_j_* are projected in a high-dimensional Hilbert space, where the mapping function is easier to retrieve. A kernel function *K*(**x***_i_*, **x***_j_*) allows to handle only inner products between pattern pairs, disregarding the specific mappings of individual patterns. The kernel trick allows setting up the non-linear variant of virtually any algorithm that can be formalized in terms of dot products.

Regularized Least Square (RLS) [34] and Support Vector Machines (SVM's) are very popular implementations of such kernel machines [31]. Both techniques belong to the class of regularized kernel methods. Thus, they identify the *f*(**x**) that best approximates *γ* by exploiting a cost function in which a positive parameter, λ, rules the tradeoff between the empirical error and a regularizing term. The decision function *f*_RLS_(**x**) can be formalized as follows:

(1) $$f_{\text{RLS}}\left(\text{x}\right)=\sum_{i}^{Np}\beta_{i}K\left({\text{x}}_{i},\text{x}\right)$$

where *K*( , ) is a kernel function and **β** = [*β_1_,…,β_Np_*] is a vector of scalar coefficients; **β** can be obtained as follows:

(2) $$\text{β}={\left(\text{K}+\lambda \text{I}\right)}^{-1}\text{y}$$

where λ is the regularization parameter, and **K** is the matrix of kernel functions *K*(**x***_i_*, **x**). In the case of SVMs, the decision function *f*_SVM_(**x**) is given by:

(3) $$f_{\text{SVM}}\left(\text{x}\right)=\sum_{i}^{\text{Nsv}}\alpha_{i}y_{i}\text{K}\left({\text{x}}_{i},\text{x}\right)+b$$

where the number of support vectors *N_sv_*, the “bias” term *b*, and coefficients *α*_i_ are computed by the training algorithm, which minimizes a quadratic cost function [31]. The eventual generalization performance of a SVM also depends on the setting of a scalar parameter, *C*, which rules the trade-off between accuracy and complexity in the training process, and actually plays the role of 1/λ [31].

The theoretical framework presented in [20] showed that the above formalism can be fruitfully applied to the sensor-based domain, and introduced a kernel function for developing tensor-based models. This result is noteworthy in that it allows every kernel machine to deal with tensors, provided that the kernel function proposed in [20] is used. This in turn means that both the kernel methods discussed above can be extended to tensor-based learning. For the sake of repeatability, the Appendix provides the procedure to handle sensor data and represent them in a tensor-based framework, for further processing within a kernel-based paradigm.

### 3.2. Applying the ML-Based Framework to the Recognition of Touch Modalities

The proposed framework tackles the interpretation of touch modalities by adopting a classification scheme that exploits tensor-based kernel methods. The ML approach splits the pattern-recognition problem into two tasks:

(1) The definition of a suitable descriptive basis for the input signal provided by the sensor (or sensor array), *i.e.*, a tensor-based description 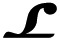 ∈ 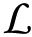, where 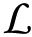 is a tensor space:
  (4) L=ϕ(S)
  In Equation (4), 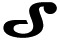 is the 3rd order tensor that characterizes sensor outputs, and the process *ϕ* works out a tensor-based description from 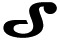, thus preserving the structure of the signal originally provided by the tactile sensor.
(2) The empirical learning of a model for the non-linear function, *γ* that maps the feature space, *F*, into the set of tactile stimuli of interest:
  (5) L→T

In principle, the learning system in Equation (5) could be designed to receive as input the tensor 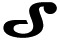 directly. However, a pre-processing may be needed to better characterize the underlying tactile phenomenon. In this regard, one should take into account that the pre-processing *ϕ* should satisfy 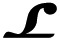 ∈ ℜ*^l^*^(1)^ ⨂ ℜ*^l^*^(2)^ ⨂ ℜ*^l(3)^*, where *l*(1), *l*(2), and *l*(3) are pattern-independent quantities.

Accordingly, the proposed ML scheme models the mapping function *γ* by using a dataset **X** holding *N_p_* patterns (samples), where each pattern includes a data tensor 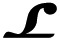 and its category label *y* ∈ {−1, 1}. Even though T usually includes several tactile stimuli and a multiclass problem is addressed, this paper aims to evaluate the advantages of introducing a tensor-based approach; hence a binary classification problem is considered without loss of generality. The literature indeed provides several effective strategies to tackle a multiclass classification scheme by integrating binary classifiers [35].

The setting of the machine adjustable parameters affects the generalization ability of a machine-learning model, *i.e.*, its ability to attain a reliable accuracy of previously unseen patterns. Indeed, the training phase is usually supported by a *model-selection* procedure, which is designed to estimate the parameter setting that may yield the most effective generalization ability. Such crucial aspect will be addressed in Section 4. In the case of tensor-SVM, three parameters are involved. The first parameter is the quantity *C*, which characterizes the learning model itself (see Section 3.1). The remaining parameters characterize the kernel function *K* described in the Appendix: the width *σ* of the Gaussian kernel, and the number of columns *α* in the matrices ${\text{V}}_{i\left(z\right)}^{\left(\alpha \right)}$ and ${\text{V}}_{j\left(z\right)}^{\left(\alpha \right)}$. The last two parameters obviously are also involved in the configuration settings of tensor-RLS; in this case the configuration set is completed by the regularization parameter *λ*, as per Equation (2).

Although a specific value of *σ* and *α* parameters can be set for each factor kernel *k*^z^, the present research adopts one *σ* value and one *α* value for every *k*^z^. The role of the latter parameter is crucial because *α* is the only quantity that is not included in the configuration of a conventional kernel machine. As anticipated above, a conventional choice for this parameter is *α* = *Q_z_*∀*z* ∈ {1, …, *Z*}. On the other hand, one should also consider that SVD can effectively take out redundancy or noise from data, and this property may prove appealing in the pattern-recognition application at hand. Thus, by fine tuning *α* one can expect to decrease the quantity of noise that affects the tensor patterns (or, more precisely, the unfolding of the tensors themselves), and in turn boost the generalization ability. As a result, in the present research the *α* quantity is treated as a configurable parameter whose value can vary between 1 and *Q_z_*.

## 4. Effective Model Selection to Boost Generalization Performance

### 4.1. The Problem of Effective Model Selection

The specific problem of interpretation of touch modalities poses major challenges to inductive learning methodologies, which induce a general rule from a set of observed instances. In fact, a relevant constraint is that the training set (*i.e.*, the observed instances) actually conveys reliable information about the unknown general rule. In the case of interpretation of touch modalities, though, the setup of such training set may not represent a straightforward task, due to the impossibility of collecting training data that are not affected by the subjective nature of the interpretation of a predetermined ‘abstract’ touch modality. For example, the same touch modality may generate stimuli that differ in the amount of pressure applied and in the length of the time window spanned by the gesture. Eventually, one cannot avoid the presence of a level of overlap between stimuli that in principle generate from different touch modalities.

The presence of noise in the training data is obviously a problem that learning machines should cope with. Indeed, the interpretation of touch modalities represents an applicative scenario in which such problem may prove critical. In this sense, the main concern is the generalization ability of the pattern-recognition system (determined by the settings of the corresponding model parameters), *i.e.*, its ability to correctly classify patterns that were not included in the training set. The accuracy at predicting unseen data is the practical criterion to evaluate the effectiveness of a trained system. Hence, the final goal of a training procedure is to define the machine parameterization that can lead to the most effective generalization performances; this process is usually named model selection. In fact, estimating the generalization performance of a learning machine is not a straightforward task. In principle, the literature [31] provides a variety of theoretical criteria to bound the generalization error of a ML system, but these approaches often lack in practicality. On the other hand, one may exploit empirical criteria [36], which use a subset of training data to support the estimation of the generalization performance. However, the empirical estimation of the generalization error may prove difficult in the presence of limited training set or in the presence of noisy data. Indeed, applications that deal with the interpretation of tactile data may suffer from both problems, as: (1) collecting training data can be onerous and (2) it is difficult to remove noise from this kind of experiments.

In the conventional formalization, the “true” generalization error, π, of a classifier is unknown because one cannot predict the classifier's behavior over the entire distribution of data; therefore, one uses the performance on the empirical training set as an estimate of π, and bounds the associate generalization performance by means of statistical penalty terms:

(6) π≤v+χ+τ

where ν is the error scored on the empirical training set, χ measures the complexity of the space of classifying functions, and τ penalizes the finiteness of the training set. In general, the task of computing χ may prove quite difficult, as the notion of “complexity” is not standard.

### 4.2. Conventional Approaches to Model Selection

The literature provides a certain variety of methods for the analytical estimation of a classifier's generalization ability; most approaches derive a bound for implementing Equation (6) by taking into account the degrees of freedom in the classifier adjustable parameters, and the configuration of the space of admissible functions that the classifier may take upon [37,38]. These methods involve a profound theoretical formalism and exhibit general applicability; nonetheless, due to the general assumptions in the classifier characterization, they mostly fail in deriving bounds that have some practical value.

On the other hand, empirical approaches to the estimation of generalization performance prove effective in practical domains, and are therefore widely adopted in real-world applications. Cross-validation [37,39] represents a popular option toward that end; the rationale of this approach is to estimate the error, π, by using available data to mimic the overall training problem. In practice, one splits the available data into a training set, used to minimize ν by adjusting the machine parameters, and a test set, which does not enter the training process and is only used to measure the actual prediction of π In order to minimize any biasing from the random-splitting process, one iterates the entire procedure in several independent runs, and computes the eventual estimated value by some statistical descriptor (e.g., average, minimum, maximum, *etc.*). One typically retains the “best” classifier, that is, the parameter settings that yielded the smallest predicted error. Although that procedure is quite popular in the literature, it might yet suffer from some statistical biasing, since the test-set performance often drives the choice of the implemented classifier, and thus enters the training process, albeit in an indirect manner.

The research presented here, therefore, adopts a more rigorous approach, involving the splitting into three independent data sets: a “training” set, a “cross validation” set (having the same meaning and purpose described above), and a “test” set, which is taken into account only after selecting the target classifier, and is used to predict the machine's generalization ability. Iterating this procedure over several independent runs removes any randomness sampling influence. This procedure will be adopted in the experimental verification of the touch-modality recognition.

An issue of these methods might consist in the fact that the empirical methods do not take into account the actual capabilities of the classifier model that is being trained, and only rely on the iterated training process for scanning the space of admissible functions. Integrating both the empirical sample and the theoretical model of the classifier yields a more accurate estimate of the generalization ability.

### 4.3. Enhancing Model Selection by Maximal-Discrepancy Method

The analysis described in [40] showed that the estimate of π in Equation (6) can be improved by exploiting the notion of complexity formalized in the Maximal Discrepancy (MD) framework [40] to assess χ. Given a training set, a classifier, and a classifier's parameterization, the MD framework estimates χ by exploiting the quantity *ν̄*; *ν̄* represents the average error scored by the classifier on *N* artificial datasets obtained by randomly swapping each time half of the labels in the original training set. Eventually, one sets χ = 1−2 *ν̄*; therefore, the complexity χ is high if the classifier can learn noise. In fact, a complex classifier is usually prone to overfitting [36], *i.e.*, an effective performance on the data included in the training set but a poor performance when processing unseen data. Thus, a highest representation capability may also lead the classification machine to model noise.

In [41], the authors indeed showed that the ability of the MD framework to estimate complexity could be further improved. In particular, the methodology discussed in [41] proved that a more accurate estimate of χ can be achieved by taking as reference the level of complexity reached by the classifier when tackling the problem represented in the original training set (*i.e.*, the complexity reached to score the training error ν). As a result, the quantity *ν̄* should be assessed by using classifiers that do not show a complexity greater than the “reference” complexity. A convenient procedure to estimate the reference complexity is given in [41]; it requires to compute two quantities:

- the hyperplane **β** ^(RKM)^ that separates the two classes (“+1” and “−1”) of the dataset **X** according to a classifier based on a regularized kernel machine;
- the hyperplane **β** ^(REF)^ that one obtains by a unsupervised evaluation of the dataset **X**.

The latter quantity can be actually computed by adopting a two-step process:

(1) divide the dataset **X** into two clusters by using an unsupervised clustering method; let X^(^*^a^*^)^ denote the subset of data assigned to the first cluster, and X^(^*^b^*^)^ denote the remaining subset of data assigned to the second cluster.
(2) obtain the hyperplanes **β** ^(+)^ and **β** ^(−)^ as follows:
  a. assign the artificial label “+1” to the data belonging to X^(^*^a^*^)^, and the artificial label “−1” to the data belonging to X^(^*^b^*^)^; apply a conventional training to this problem to obtain the hyperplane **β** ^(+)^ that separates the two classes.
  b. Assign the artificial label “−1” to the data belonging to X^(^*^a^*^)^, and the artificial label “+1” to the data belonging to X^(^*^b^*^)^; apply a conventional training to this problem to obtain the hyperplane **β** ^(−)^ that separates the two classes.
  c. Set **β** ^(REF)^ as follows
    (7) $${\text{β}}^{\left(\text{REF}\right)}=\underset{\text{w}}{\arg\min}\left(\left\|{\text{β}}^{\left(+\right)}-{\text{β}}^{\left(\text{RKM}\right)}\right\|,\left\|{\text{β}}^{\left(-\right)}-{\text{β}}^{\left(\text{RKM}\right)}\right\|\right)$$

**Figure 4.**
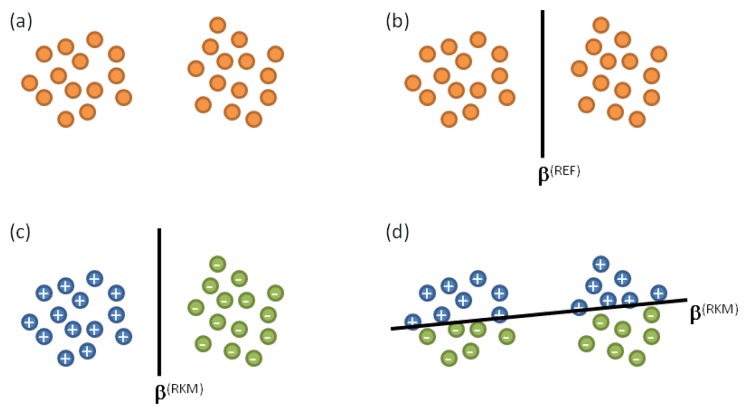
Difference between natural distribution of data and true distribution of data.

The rationale behind this approach can be explained by analyzing the configuration schematized in Figure 4. Figure 4a proposes a problem in which the data belonging to **X** are intrinsically organized in two clusters. Thus, the unsupervised evaluation of the dataset would lead to the situation illustrated in Figure 4b, which reports the approximate position of the hyperplane **β**^(REF)^. One may conclude that **β**^(REF)^ characterizes the “natural” distribution of data. On the other hand, the position of the hyperplane **β**^(RKM)^ would result from the analysis of the empirical data; *i.e.*, **β**^(RKM)^ is obtained by taking into account the actual labels associated to each pattern. As a result, two different situations may arise from the proposed example. Figure 4c refers to the first situation, in which it is supposed that clusters match the actual classes. Conversely, Figure 4d refers to the opposite situation, in which it is supposed that actual classes do not match the natural distribution of data. In the case of Figure 4c, **β**^(REF)^ ≡ **β**^(RKM)^; in the case of Figure 4d, **β**^(REF)^ ∈ **β**^(RKM)^. Therefore whenever the result of clustering matches the true distribution of pattern classes, the unsupervised separation surface **β**^(REF)^ and the real classification surface must coincide **β**^RKM)^. Of course, the opposite case may occur, in which the target distribution is totally uncorrelated with the obtained clusters. In general, however, **β**^(REF)^ and **β**^(RKM)^ set the constraints for the admissible solutions to the classification problem at hand, as proved in [41]. As a major consequence, such constraints should represent the reference when assessing χ by adopting the MD framework.

For the sake of clarity, Algorithm 1 reports the full procedure to assess χ. In the case of the present framework, the tensor-based versions of SVM and RLS are the classification tools that support the interpretation of touch modalities. Therefore, model selection is designed to set the best parameterization for those machines. The tensor-based version of the kernel k-means clustering method provided the unsupervised tool to be used in the model selection procedure.


**Algorithm 1** Complexity Assessment
Input:training set **X**= {(

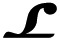
, *y*)*_i_*; *i* = 1,..,*N_p_*},kernel parameters *σ* and *α*regularization coefficient *λ*scaling coefficient εOutput:estimated complexity χI.*Compute the kernel*
Build the kernel matrix **K** on data { 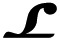; *i* = 1,..,*N_p_*} with parameters *σ* and *α*II.*Unsupervised Clustering*
Divide the data { 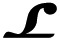; *i* = 1,..,*N_p_*} into two clusters by exploiting kernel-kmeans with kernel **K**Denote with X^(^*^a^*^)^ and X^(b)^ the two clustersIII.*Compute*
**β**^(RKM)^
Train the RC on the original training set **X: β**^(RKM)^ = RCtraining(*λ*, **K**, *y*)IV.*Compute***β**^(+)^
Apply an artificial labeling schema: X^(^*^a^*^)^→*y* = +1, X^(^*^b^*^)^→*y* = −1Train the RC on the dataset X^(^*^a^*^)^∪ X^(^*^b^*^)^ with kernel **K**: **β**^(+)^ = RCtraining(*λ*, **K**, *y*)V.*Compute*
**β**^(-)^
Apply an artificial labeling schema: X^(^*^a^*^)^→*y* = +1, X^(^*^b^*^)^→*y* = −1Train the RC on the dataset X^(^*^a^*^)^∪ X^(^*^b^*^)^ with kernel **K**: **β**^(-)^ = RCtraining(*λ*, **K**, *y*)VI.*Set reference*
Set Γ_0_ = **β**^t^**Kβ**where: $\text{β}=\underset{\text{w}}{\arg\min}\left(\left\|{\text{β}}^{\left(+\right)}-{\text{β}}^{\left(\text{RKM}\right)}\right\|,\left\|{\text{β}}^{\left(-\right)}-{\text{β}}^{\left(\text{RKM}\right)}\right\|\right)$VII.*Compute v̅*
Set ν̄ = 0for *i* = 1 to *N*
Set *λ̂* = *λ*Generate an artificial training set X* = {( 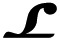, y*)*_i_*; *i*=1,..,*N_p_*}, where y* is obtained by randomly swapping half of the label in *y*Train the RC on X*
**β** = RCtraining(*λ̂*, **K**, y*)Set Γ^*^ = β^t^KβCompute the classification error ν* on X*if (Γ^*^ > Γ_0_) then: set *λ̂* = ε·*λ;* goto 2.c;*ν̄* = *ν̄* + ν*Set χ = 1 − 2 *ν̄*/*N*


## 5. Results and Discussion

### 5.1. Dataset and Preprocessing

The 70 participants involved in the experiments were required to touch the outer surface of the sensor array using three reference actions (= modalities of touch): sliding the finger, brushing a paintbrush and rolling a washer. The corresponding outputs of the sensor array were collected to build the dataset used as benchmark to test the proposed framework. Not to influence the participant and to allow his/her subjective gesture interpretation, each person was given a written protocol as a guide for the experiments. No particular indications were given to the participants about the duration of the stimuli and the pressure level to apply (the only constraint was to complete every single touch within a time window of 7 s).

For each reference action, every participant was asked to first touch the sensor array moving horizontally over a random line, then repeating the action over a randomly chosen vertical line (two different acquisitions). The participant was therefore asked to repeat the six experiments in the same order to get a second sampling. This is because the first sampling was intended as practicing the imagined gesture by touching the real skin, therefore enabling *a* “*more spontaneous and natural behavior*” for the more *aware* second sampling.

The number of acquired patterns (each pattern consists of 16 time signals corresponding to *charge response vs. time* provided by each sensor building the sensor array) was 840 (70 participants, three modalities, four patterns for each modality—*i.e.*, horizontal and vertical gestures, two runs each). Half of these patterns were actually used in the pattern-recognition analysis, corresponding to the second sampling by each participant and ensuring more spontaneous behavior.

The collected patterns were expressed by a 3-dimensional tensor, 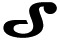 ∈ ℜ^4^ ⨂ ℜ^4^ ⨂ ℜ^21000^. The extension of the 3rd component was determined by the time window allowed in each experiment (7 s) and the adopted sample rate (3 ksps). In fact, when applying the tensor-based kernel approach to those original signals, one is expected to work out the SVD of a matrix having 21,000+ elements in one of its dimensions; such a computationally impractical task would prove ineffective in terms of numerical accuracy. As a consequence, the pre-processing *ϕ* discussed in Section 3.2 remapped the original tensor mostly to reduce the dimensionality of the 3rd component of 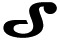.

The implementation of *ϕ* adopted in this work was designed to take into account two main issues. First, only a limited portion of the 21,000 elements in the 3rd component of 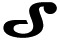 actually carry information about the tactile stimulus: for any pattern, the signal of interest lies within a limited time window, whose width depends on the pattern itself. Secondly, the preprocessing, *ϕ*, should satisfy: 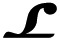 = *ϕ*( 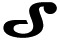), with 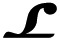 ∈ ℜ^4^ ⨂ ℜ^4^ ⨂ ℜ*^l^*^(3)^, where *l*(3) is a pattern-independent quantity.

**Figure 5.**
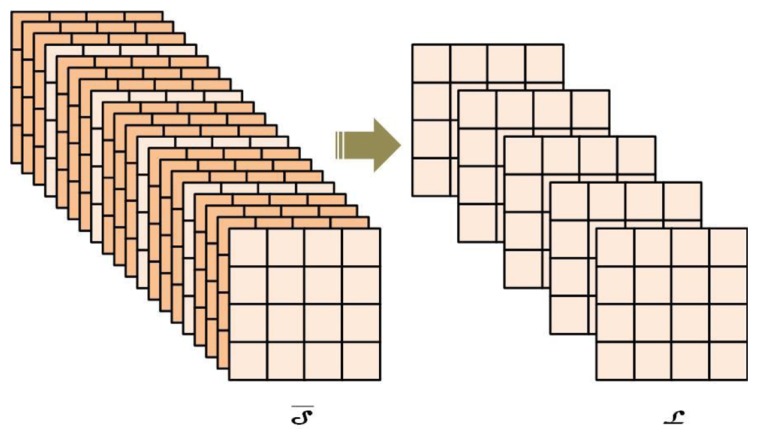
A schematization of the pre-processing strategy based on sub-sampling.

The localization of the relevant time window in the 3rd component of 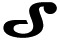 was obtained by analyzing the amount of energy provided by the single elements of the sensor. In the following, for the *i*-th pattern, ${\bar{S}}_{i}$ is the 3rd-order tensor obtained after extracting the relevant time window from 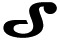
_i_; thus 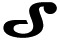
_i_ ∈ ℜ^4^ ⨂ ℜ^4^ ⨂ ℜ*^Si^*. Then, Algorithm 2 was adopted to shrink the 3rd component of ${\bar{S}}_{i}$. In this algorithm (Figure 5), a subsampling strategy is applied to work out 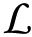
_i_ from ${\bar{S}}_{i}$. The tensor ${\bar{S}}_{i}$ and the expected size *D* of the 3rd component of 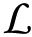
_i_ are the algorithm inputs.


**Algorithm 2** Data Pre-processing
Input:tensor 
$\bar{S}$parameter *D*Output:tensor **L***Compute the sampling interval*  = └*S_i_* / *D*┘*Obtain*
***L****p* = 1for *d = 1,..,D***L**_(:,:_,*_d_*_)_ = 
$L_{\left(:,:,d\right)}={\bar{\text{S}}}_{\left(:,:,p\right)}$***p* = *p* +**


### 5.2. Experiments: Binary Classification

The effectiveness of the pattern-recognition system in the classification of touch modalities was evaluated by using the dataset obtained as per Section 5.1. The final dataset only covered 65 out of the original 70 participants to remove apparent outliers or extremely noisy results, and only the patterns collected in the 2nd run from each participant entered the pattern-recognition simulations. The experimental session involved three binary classification problems:

- “brushing a paintbrush” *versus* “rolling a washer”;
- “brushing a paintbrush” *versus* “sliding the finger”;
- “rolling a washer” *versus* “sliding the finger”.

The dataset included—for each modality and for each participant—both the horizontal and the vertical gestures, thus each binary testbed held 260 patterns (2 modalities × 65 participants × 2 gestures). The generalization performance of the ML-based classification was measured by randomly splitting the dataset into a training set and a validation set, holding 180 patterns and 80 patterns, respectively. The former drove the adjustment of the classifiers parameters, thus supporting model selection. The latter was used to measure classification accuracy on unseen data (*i.e.*, an empirical estimation of the term π in Equation (6)). The two sets never shared any participant; this made it possible to estimate the generalization ability of the ML algorithm with respect to unseen users, as well. To provide statistical robustness in the generalization estimates, the splitting process was iterated over five different training/validation pairs (*i.e.*, five different, independent runs were completed).

The following settings were adopted for the three kernel parameters that determined the generalization performances of the tensor-based classifiers:

- *λ* ∈ {10^−3^, 10^−2^, 10^−1^, 10^0^, 10^1^};
- σ ∈ {2^−4^, 2^−3^, 2^−2^, 2^−1^, 2°, 2^1^, 2^2^, 2^3^, 2^4^};
- α ∈ {1, *Q_z_*/2, *Q_z_*}.

Parameter *C* in SVM plays the role of 1/*λ*, where *λ* is the quantity that rules the trade-off between the empirical error and a regularizing term (as per Section 3.1). Parameter σ characterizes the specific kernel function adopted in this work (see Appendix). The options for the parameter α indicate three options to the removal of columns from matrixes **V**_(_*_z_*_)_ in the kernel computation (see Appendix): setting α = 1 meant that only the column associated with the largest singular value was retained; when α = *Q_z_*/2, half of the significant columns were kept, whereas setting α = *Q_z_* implied that no column was removed from **V**_(_*_z_*_)_. Overall, the model selection procedure aimed to pick out the most effective setup from among the various available configurations. In each run, the procedure described in Algorithm 1 processed the training set and identified the parameter values yielding the best predicted performance. Eventually, the performance on the validation set gave an estimate of the actual generalization error, π, thus measuring the relative effectiveness of the model selection procedures.

Tables 1, 2 and 3 give the simulation results for the classification problems A, B, and C, respectively. Each table reports on the performance attained on the associate problem when applying the tensor-SVM model. The experiments involved three settings for parameter *D*, which drove the sub-sampling rate in the pre-processing approach as per Algorithm 2: *D* ∈ {20, 50, 100}. As a result, for each pair (run, *D*), the table gives:

- the classification error percentage attained on the validation set by the ML-based predictor;
- the parameters setting {*λ*, σ, α} used in the predictor as a result of the model selection procedure.

**Figure 6.**
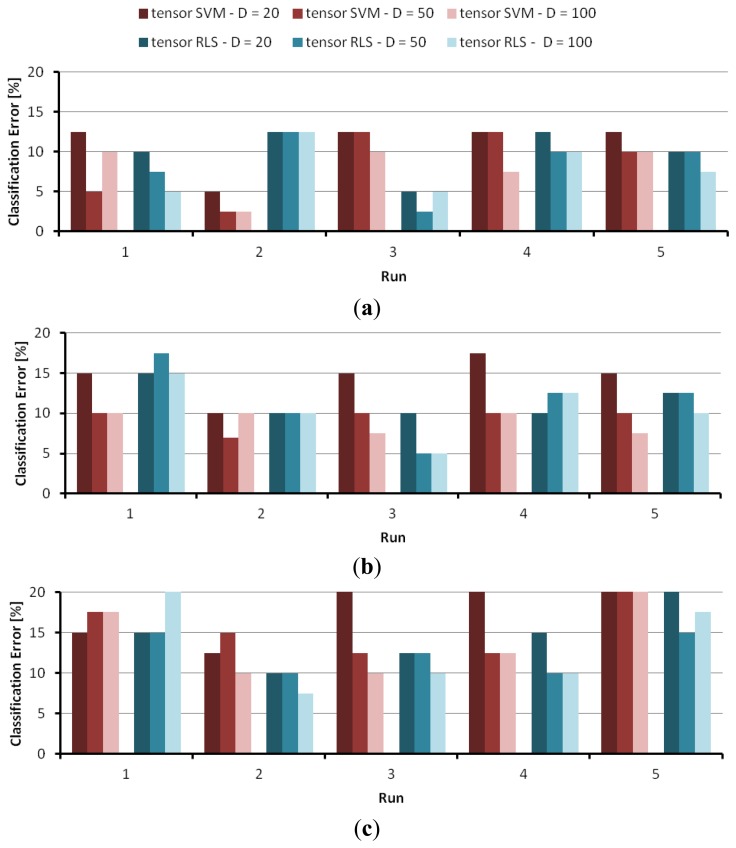
Results obtained with tensor-SVM and tensor-RLS for the classification problems A, B, C: (**a**) problem A; (**b**) problem B; (**c)** problem C.

Likewise, Tables 4, 5 and 6 report on the simulation results obtained by using the tensor-RLS predictor model. The graphs in Figure 6 recap visually the table results, and provide a chart for each classification problem. In each chart, the *x* axis marks the five runs, and the *y* axis gives the classification error on the validation set. For each run, six values are plotted: the classification errors attained by tensor-SVM @ *D* = {20, 50, 100}, and the classification errors attained by tensor-RLS @ *D* = {20, 50, 100}.

Empirical evidence proves that the tensor-based pattern-recognition technologies could effectively support the classification problems. An analysis of numerical results leads deriving some remarks. First, accuracy values confirmed that the touch-modality recognition problem involved a challenging task. In some ways, this might be ascribed to the protocol for data collection, which was designed to avoid specific constraints on the participants' behavior but ultimately widened the variance in empirical data. On one hand, the protocol ensured that gestures were spontaneous and natural; on the other hand, this inevitably induced a level of overlap between stimuli that in principle belonged to different touch modalities

**Figure 7.**
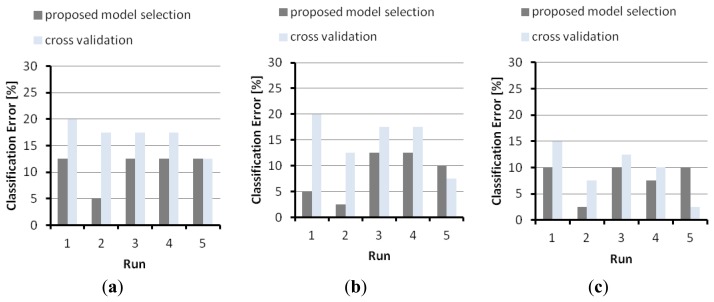
A comparison between the generalization performance obtained by applying the model selection of Algorithm 1 and conventional cross-validation. The graphs refer to problem A, tensor-SVM: (**a**) *D* = 20; (**b**) *D* = 50; (**c**) *D* = 100.

The core of the research presented in this paper consists in a practical approach to the model-selection problem for effective parameter setting in real applications. Toward that purpose, the graphs in Figure 7 highlight the advantages of the Maximal-Discrepancy criterion to model selection, and compare the model selection performed according to Algorithm 1, and the model selection resulting from conventional cross-validation. In the latter tests, the training set including 180 patterns was repeatedly split into a training and a test set for model selection, and the remaining 80 patterns formed the validation set for unbiased error estimate; the same validation set was used to evaluate the generalization error scored by the former method under MD-based selection. For the sake of brevity, Figure 7 only refers to problem A, but similar results were observed for all problems. The graphs give the classification errors (on the common validation set) by the tensor-SVMs. Figures 7 refer to the experiments with *D* = 20, *D* = 50, and *D* = 100, respectively. In each graph, the *x* axis marks the five different runs, whereas the *y* axis gives the classification error (error percentage on the validation set).

**Figure 8.**
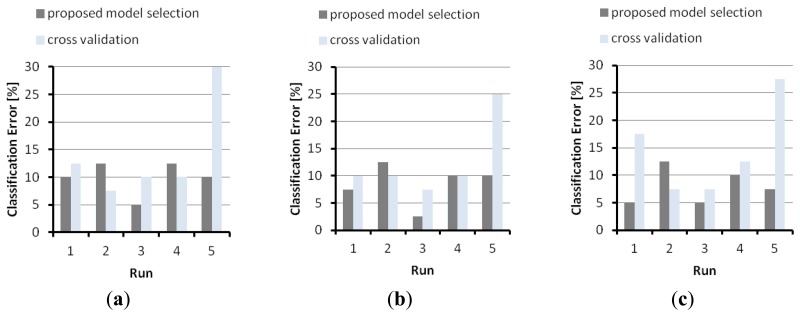
A comparison between the generalization performance obtained by applying the model selection of Algorithm 1 and conventional cross-validation. The graphs refer to problem A, tensor-RLS: (**a**) *D* = 20; (**b**) *D* = 50; (**c**) *D* = 100.

Figure 8 illustrates the results obtained on problem A with tensor-RLS. The graphs again prove that model selection supported by Algorithm 1 mostly yielded lower validation errors than those achieved by conventional cross-validation.

Secondly, numerical results seem to suggest that–overall–tensor-SVM slightly outperformed tensor-RLS on the various classification problems. However, one should consider that, in both cases, the classification error scored on a specific classification problem varies significantly across the different runs. This behavior actually confirms that the presence of noise and variance may be a major concern when dealing with tactile data.

Finally, classification problem C proved to be the most difficult task for the ML systems, as the predictors sometimes could not attain a lower classification error than 20%. Such a result indicates that the involved touch modalities (“sliding the finger” and “rolling a washer”) proved quite difficult to discriminate. Conversely, the best performances in terms of classification error were obtained for problem A, in which tensor-SVM scored a classification error of 2.5%.

### 5.3. Experiments: Multiclass Problem

A second empirical session addressed a 3-class classification problem, involving the touch modalities covered by the dataset. Three “1-versus-all” predictors were independently trained to solve as many binary classification problems (“one touch modality *versus* the others”); as a result, each predictor could yield two alternative results: either the test pattern was ascribed to the classifier-specific touch modality, or the classifier prompted a “don't know” outcome. The system finally assigned a touch modality to a test pattern according to the following rules:

(1) if one classifier ascribed the test pattern to a specific touch modality, whereas the other modules both prompted a “don't know” outcome, the pattern was classified accordingly;
(2) otherwise, the pattern was categorized according to the predictor whose decision function, *f*(**x**), turned out to be highest.

The multiclass experiment was set up according to the following algorithm:

(1) Randomly split the set of 65 participants into two subsets, TG and TT, including 45 participants and 20 participants, respectively.
(2) For each 1-vs-all classification problem, generate a training set containing a total of 180 patterns. Half of the patterns are gathered by including –for each participant in TG—the horizontal and the vertical gestures associated to the touch modality addressed by the specific classification module (45 participants × 2 gestures = 90 patterns). The remaining 90 patterns are obtained by randomly selecting—for the participants in TG—gestures associated to the other two touch modalities.
(3) Generate a test set by including -for each modality and for each participant in TT- both the horizontal and the vertical gestures. As a result, the test set holds (3 modalities × 20 participants × 2 gestures =) 120 patterns.

To ensure statistical robustness in the generalization estimates, the splitting/training/testing process was iterated over five different runs. Table 7 gives the simulation results for the classification problem. The table reports on the performance attained with the tensor-SVM and the tensor-RLS. Two quantities measure overall performances: (1) the best classification error attained on the test set over the five different runs; (2) the classification error on the test set, averaged over the five different runs. In the table, the last row points out the parameter settings that yielded the reported performances.

The results reported in Table 7 show that the tensor-based method was able to address the 3-touch classification problem. Measured performances, though, confirmed that the recognition of touch modalities did prove a challenging task, as one should deal with classification errors that are larger than 22%. As anticipated above, such results should be analyzed by taking into account the protocol adopted for data collection.

## 6. Conclusions

This paper addressed the development of computational intelligence techniques to recognize touch modalities using an artificial skin as sensor system. The proposed pattern-recognition system is specifically designed to deal with the tensor morphology of the tactile signals.

A dedicated experimental campaign, involving a high number of participants, gave a representative tactile data set, e.g., a wide range of interpretations of the experimental protocol. The reported results prove that the proposed pattern-recognition system achieves consistent performance on the bi-class classification problems adopted as test bed. In this regard, the paper introduced a framework that embedded a criterion to drive model selection effectively, and therefore supported practical applications involving tactile interaction problems.

The paper focused on the discrimination of touch modalities, but it is worth noting that a tensor-based approach might be useful in general for discriminating tactile data. When extending the framework to a wider range of tactile data, the major benefits would certainly consist in the specific tensor-based representation, which best fits the nature of empirical data, and in the capability of Machine Learning tools to acquire classification procedure in an automated and empirical way. On the other hand, some drawbacks might derive from the need for adequate and effective features to express the mission-critical data contents, and in the requirement of a considerable amount of empirical observations to avoid or limit over-fitting phenomena.

A fair comparison with other ML-based approaches to the classification of touch modalities [13–15] may prove difficult to carry out because of both the lack of a common test bed and the dissimilarity in the prescribed targets. Actually, it is worth noting that the approaches proposed in the literature exploited ML technologies without addressing explicitly the issue of model parameterization, hence the specific contribution of those works consists in showing that ML tools such as SVM, SOM, AdaBoost can tackle effectively the interpretation of touch modalities. On the other hand, those methods do not cover the problem of model selection and its related outcomes, which is instead the core of the framework tackled in this paper.

Predicting generalization performance is in fact extremely important, in that every learning machine is characterized by a set of adjustable parameters. The design of an effective classifier requires that, first, a criterion to drive model selection should always be defined, and, second, generalization performance is evaluated only after the model parameters have been set for run-time operation. This is especially true in the presence of complex domains when few empirical data are available, as is the case of touch-modality recognition.

From this viewpoint, the paper has confirmed the complexity of the underlying sensorial problem, but, on the other hand, the research yielded a reliable and practical procedure to predict a system performance before deployment. A comparison of the Maximal-Discrepancy method with conventional cross-validation supported the advantages of the former approach in the tensor-based paradigm.

## Figures and Tables

**Table 1.** Simulation results: problem A, tensor-SVM.

	*D*		

	20	50	100
run #1	12.5 (0.1, 2^1^, *Q_z_* /2)	5.0 (10, 2^3^, 0)	10.0 (1, 2^1^, 0)
run #2	5.0 (0.1, 2^1^, 0)	2.5 (1, 2^1^, 0)	2.5 (1, 2^1^, 0)
run #3	12.5 (0.1, 2^2^, *Q_z_* /2)	12.5 (1, 2^2^, *Q_z_*)	10.0 (0.1, 1,*Q_z_* /2)
run #4	12.5 (0.1, 2^1^, 0)	12.5 (0.1, 1, *Q_z_* /2)	7.5 (0.1, 2^−1^, 0)
run #5	12.5 (1, 2^1^,*Q_z_*)	10.0 (10, 2^3^, 0)	10.0 (1, 2^1^, 0)

**Table 2.** Simulation results: problem B, tensor-SVM.

	*D*		

	20	50	100
run #1	15.0 (0.1, 1, *Q_z_*/2)	10.0 (0.1, 2^−1^, *Q_z_*/2)	10.0 (0.1, 2^−1^, *Q_z_*/2)
run #2	10.0 (0.1, 2^−2^, *Q_z_*/2)	7.0 (0.1, 2^−2^, *Q_z_*/2)	10.0 (0.1, 2^−1^, *Q_z_*/2)
run #3	15.0 (0.1, 1, *Q_z_*/2)	10.0 (0.1, 1, *Q_z_*/2)	7.5 (0.1, 1, *Q_z_*/2)
run #4	17.5 (1, 2^2^, *Q_z_*/2)	10.0 (0.1, 2^−1^, *Q_z_*/2)	10.0 (0.1, 2^−1^, *Q_z_*/2)
run #5	15.0 (1, 2^−1^, *Q_z_*/2)	10.0 (1, 2^2^, *Q_z_*/2)	7.5 (1, 2^2^, *Q_z_*/2)

**Table 3.** Simulation results: problem C, tensor-SVM.

	*D*		

	20	50	100
run #1	15.0 (0.1, 1, *Q_z_*/2)	17.5 (0.1, 1, *Q_z_*/2)	17.5 (0.1, 1, *Q_z_*/2)
run #2	12.5 (1, 2^4^, *Q_z_*/2)	15.0 (10, 2^3^, *Q_z_*/2)	10.0 (1, 2^−1^, 0)
run #3	20.0 (0.1, 2^1^, *Q_z_*/2)	12.5 (1, 2^2^, *Q_z_*/2)	10.0 (1, 2^2^, *Q_z_*/2)
run #4	20.0 (1, 2^2^, *Q_z_*/2)	12.5 (0.1, 2^−1^, *Q_z_*/2)	12.5 (0.1, 2^−1^, *Q_z_*/2)
run #5	20.0 (1, 2^1^, *Q_z_*/2)	20.0 (10, 2^3^, *Q_z_*/2)	20.0 (10, 2^3^, *Q_z_*/2)

**Table 4.** Simulation results: problem A, tensor-RLS.

	*D*		

	20	50	100
run #1	10.0 (0.1, 2^2^, *Q_z_*/2)	7.5 (1, 2^3^, *Q_z_*/2)	5.0 (10, 2^4^, 0)
run #2	12.5 (1, 2^3^, *Q_z_*/2)	12.5 (1, 2^3^, *Q_z_*/2)	12.5 (0.1, 2^3^, *Q_z_*/2)
run #3	5.0 (0.1, 2^2^, 0)	2.5 (10, 2^4^, 0)	5.0 (1, 2^2^, 0)
run #4	12.5 (10, 2^4^, *Q_z_*/2)	10.0 (10, 2^4^, *Q_z_*/2)	10.0 (1, 2^3^, *Q_z_*/2)
run #5	10.0 (1, 2^4^, *Q_z_*/2)	10.0 (1, 2^3^, *Q_z_*/2)	7.5 (1, 2^4^, *Q_z_*/2)

**Table 5.** Simulation results: problem B, tensor-RLS.

	*D*		

	20	50	100
run #1	15.0 (0.1, 2^2^, *Q_z_*)	17.5 (0.1, 2^3^, *Q_z_*)	15.0 (0.1, 2^2^, *Q_z_*)
run #2	10.0 (10, 2^2^, *Q_z_*/2)	10.0 (10, 2^1^, *Q_z_*/2)	10.0 (10, 2^1^, *Q_z_*/2)
run #3	10.0 (0.1, 2^2^, *Q_z_*/2)	5.0 (1, 2^3^, *Q_z_*/2)	5.0 (0.1, 2^2^, *Q_z_*/2)
run #4	10.0 (1, 2^4^, *Q_z_*)	12.5 (1, 2^4^, *Q_z_*)	12.5 (1, 2^4^, *Q_z_*)
run #5	12.5 (1, 2^3^, *Q_z_*/2)	12.5 (1, 2^3^, *Q_z_*/2)	10.0 (1, 2^3^, *Q_z_*/2)

**Table 6.** Simulation results: problem C, tensor-RLS

	*D*		

	20	50	100
run #1	15.0 (1, 2^4^, *Q_z_*/2)	15.0 (1, 2^4^, *Q_z_*/2)	20.0 (0.1, 2^4^, *Q_z_*/2)
run #2	10.0 (1, 2^3^, 0)	10.0 (1, 2^3^, *Q_z_*/2)	7.5 (1, 2^4^, 0)
run #3	12.5 (1, 2^3^, *Q_z_*/2)	12.5 (0.1, 2^2^, *Q_z_*/2)	10.0 (1, 2^3^, *Q_z_*/2)
run #4	15.0 (0.1, 2^3^, *Q_z_*/2)	10.0 (10, 2^1^, *Q_z_*/2)	10.0 (10, 2^1^, *Q_z_*/2)
run #5	20.0 (100, 2^1^, *Q_z_*/2)	15.0 (1000, 2^3^, *Q_z_*/2)	17.5 (10, 2^2^, *Q_z_*/2)

**Table 7.** Results for the 3-class classification problem.

	Tensor-SVM	Tensor-RLS
best	23.4	22.7
average	29.0	26.3
settings	*σ* = 2^1^; *C* = 10 *α* =*Q_z_*/2; *D* = 100	*σ* = 2^-1^; *C* = 100 *α* =*Q_z_*/2; *D* = 100

## Appendix: Tensor-Based Sensorial Signal Processing

This Appendix outlines the steps to compute the entries of a tensor-based kernel function, *K*. The scheme refers to the computation of a generic entry *K*(*i*, *j*) of the complete matrix, where *i* and *j* are two patterns of the dataset. First, some notations are introduced:

- is a *Z*-th order tensor; thus, ℜ*^l(1)^* ⊗ ℜ*^l(2)^* ⊗⋯⊗ ℜ*^l(Z)^*.
- *_i_* and *_j_* are the tensors that characterize the *i*-th pattern and the *j*-th pattern, respectively.
- *K*(*i*, *j*) is the kernel entry for patterns *_i_* and *_j_*; hence, *K*(*i*, *j*) ≡ *K*( *_i_*, *_j_*).

The four steps to be completed to work out a kernel entry *K*(*i*, *j*) can be formalized as follows. For the sake of clarity, each step ends by reporting the inputs to be processed and the generated outputs.

- Unfolding
  The *unfolding* of a tensor implies to convert the elements of  to a matrix [20]. As  is a *Z*-th order tensor, *Z* matrixes are obtained by applying as many unfolding ways (or modes) [42]. Accordingly, _(_*_z_*_)_ ∈ ℜ*^Mz^* ⊗ ℜ*^Nz^* is the *z*-th mode matrix unfolding, where
  *Mz*. = *l(z)*
  *Nz*. = *l*(*z* + 1), *l*(*z* + 2) ⋯¨*l*(*Z*), *l*(1), *l*(2) ⋯·, *l*(*z* − 1).
  Inputs: *_i_* and *_j_*
  Outputs: { *_i_*_(1)_, *_i_*_(2)_,…, *_i_*_(_*_Z_*_)_} and{ *_j_*_(1)_, *_j_*_(2)_,…, *_j_*_(_*_Z_*_)_}, where *_i_*_(_*_z_*_)_, *_j_*_(_*_z_*_)_ ∈ ℜ*^Mz^*. ⊗ ℜ*^Nz^*.
- Singular Value Decomposition
  All the matrix unfoldings *_i_*_(_*_z_*_)_, *_j_*_(_*_z_*_)_ are factorized by using Singular Value Decomposition (SVD) [20]. In general, a matrix unfolding _(_*_z_*_)_ ∈ ℜ*^Mz^* ⊗ ℜ*^Nz^* yields the following factorization:
  $$L_{\left(z\right)}={\text{U}}_{\left(z\right)}{\text{S}}_{\left(z\right)}{\text{V}}_{\left(z\right)}^{t}$$
  where **U** is an *Mz* × *Mz* orthogonal matrix and **V** is an *Nz* × *Nz* orthogonal matrix. **S** is an *Mz* × *Nz* matrix whose off-diagonal entries are all zeros and whose diagonal elements satisfy
  $$s_{1}\geq s_{2}\geq \cdots \ldots ‥\geq s_{Qz}\geq 0$$
  where *Qz* = *min*(*Mz*, *Nz*). Actually, SVD allows one to obtain a matrix approximation of _(_*_z_*_)_ by removing from both **S** and **V** all the columns from the (*α* + 1)th to the *N_z_*th (thus, 1 ≤ *α* < *N_z_*).
  Inputs: { *_i_*_(1)_, *_i_*_(2)_,…, *_i_*_(_*_Z_*_)_} and{ *_j_*_(1)_, *_j_*_(2)_,…, *_j_*_(_*_Z_*_)_}
  Outputs: {**V** *_i_*_(1)_, **V** *_i_*_(2)_,…, **V** *_i_*_(_*_Z_*_)_} and {**V** *_j_*_(1)_, **V** *_j_*_(2)_,…, **V***_j_*_(_*_Z_*_)_}, where **V** *_i_*_(_*_z_*_)_,**V** *_j_*_(_*_z_*_)_ ∈ ℜ*^Nz^* ⊗ ℜ*^Nz^*
- Factor Kernel
  A factor kernel *k*^z^ is worked out for each *z*∈{1, …, *Z*}. The factor kernel is defined as:
  A1 $$k^{z}\left(i,j\right)=\exp\left(-\frac{1}{2\sigma^{2}}{\left\|{\text{V}}_{i\left(z\right)}^{\left(\alpha \right)}{\text{V}}_{i\left(z\right)}^{\left(\alpha \right)\text{t}}-{\text{V}}_{j\left(z\right)}^{\left(\alpha \right)}{\text{V}}_{j\left(z\right)}^{\left(\alpha \right)\text{t}}\right\|}_{F}^{2}\right)$$
  where ∥·∥*_F_* is the Frobenius norm. In (A1), ${\text{V}}_{i\left(z\right)}^{\left(\alpha \right)}$ is the matrix obtained by removing from **V***_i_*_(z)_ all the columns from the (*α* + 1)th to the *N_z_*th, The same notation holds for ${\text{v}}_{j\left(z\right)}^{\left(\alpha \right)}$. In principle, one can set *α* = *Q_z_* ∀*z* ∈ {1, …, *Z*}, as the remaining columns of **V**_(_*_z_*_)_ do not carry any useful information on _(_*_z_*_)_ (see Step II). However, the present work will show that *α* can be considered a tunable parameter (with *α* < *Q_z_*). Such aspect will be discussed in details in Section 3.3.
  Inputs: {**V***_i_*_(1)_, **V***_i_*_(2)_,…, **V***_i_*_(_*_Z_*_)_} and {**V***_j_*_(1)_, **V***_j_*_(2)_,…, **V***_j_*_(_*_Z_*_)_}
  Outputs: *k*^z^(*i*, *j*), with *z* = 1,..,*Z*
- Kernel Entry
  The kernel entry *K*(*i*, *j*) is finally obtained as follows:
  A2 $$K\left(i,j\right)=\prod_{z=1}^{Z}k^{z}\left(i,j\right)$$
  Expressions (A1) and (A2) show that *K*(*i*, *j*) is a product of Gaussian kernels. Thus the kernel function (A2) actually extends to tensor patterns the conventional Gaussian (RBF) kernel [31], which represents a popular choice for standard learning systems based on kernel machines.
  Inputs: {*k*^1^(*i*, *j*), *k*^2^(*i*, *j*), …, *k^Z^*(*i*, *j*)}
  Output: *K*(*i*, *j*)
